# Genomic Prediction and Genome-Wide Association Study for Growth-Related Traits in Taiwan Country Chicken

**DOI:** 10.3390/ani15030376

**Published:** 2025-01-28

**Authors:** Tsung-Che Tu, Chen-Jyuan Lin, Ming-Che Liu, Zhi-Ting Hsu, Chih-Feng Chen

**Affiliations:** 1 Department of Animal Science, National Chung Hsing University, Taichung 402202, Taiwan; tctu0523@gmail.com (T.-C.T.); foru0510@gmail.com (C.-J.L.); 2 Ray Hsing Agricultural Biotechnology Co., Ltd., Yunlin 633103, Taiwan; luimingzhe@gmail.com (M.-C.L.); qq28128qq28128@gmail.com (Z.-T.H.); 3 The iEGG and Animal Biotechnology Center, National Chung Hsing University, Taichung 402202, Taiwan

**Keywords:** genomic prediction, single-step GBLUP, genome-wide association study, marker-assisted selection, Taiwan Country chicken

## Abstract

Taiwan Country chickens are integral to Taiwanese culture and the poultry industry. By establishing a crossbreeding system, breeders must consider the growth-related traits of the dam line to achieve acceptable traits in commercial meat-type chickens. This study compared the accuracy of genomic estimated breeding values (GEBVs) predicted using the pedigree-based best linear unbiased prediction (PBLUP) model and the single-step genomic BLUP (ssGBLUP) model. Additionally, we conducted a genome-wide association study (GWAS) to identify single-nucleotide polymorphisms (SNPs) associated with growth, shank, and body conformation traits to support marker-assisted selection (MAS). The results showed that the ssGBLUP model achieved 4.3% to 16.4% higher prediction accuracy than the PBLUP model. GWAS identified four missense SNPs and four significant SNPs associated with body weight, shank length, and shank width at 12 weeks. These findings highlight the potential of integrating the ssGBLUP model with identified SNPs to improve genetic gain and breeding efficiency and provide preliminary results to assess the feasibility of genomic prediction and MAS in Taiwan Country chicken breeding programs. Further research is necessary to validate these findings and explore their mechanisms and broader application across different breeding programs, particularly for the NCHU-G101 breed of Taiwan Country chickens.

## Simple Summary

The single-step genomic best linear unbiased prediction (ssGBLUP) model improved the prediction accuracy of estimated breeding values for growth, shank, and body conformation traits by 4.3% to 16.4%. A genome-wide association study (GWAS) identified four missense SNPs and four significant SNPs, which may be useful for marker-assisted selection (MAS) in chicken breeding. Our findings contribute to further research aimed at confirming the mechanisms and long-term impacts of these SNPs and this prediction model on Taiwan Country chicken breeding programs.

## 1. Introduction

Taiwan Country chickens play an important role in Taiwanese culture and the poultry industry. These chickens are known for their distinctive meat quality and adaptability to the local environment [1,2]. However, the production system in the Taiwan Country chicken industry has been focused on selection for body weight in a “like-pure” bred population in the past decade. This system, involving continuous backcrossing without pedigree information, has led to a continuous decline in the laying performance of breeder chickens and caused instability in breed characteristics. Consequently, the egg production rate of Taiwan Country chicken breeders is significantly lower than that of broiler breeders, leading to higher production costs for commercial day-old chicks [3]. Moreover, the reliance on a purebred production system has decreased the resilience of this population to environmental stress compared to crossbred lines [4]. Given the increasing frequency of global avian influenza outbreaks and extreme climate patterns, environmental resilience has become an important trait for chicken populations [5]. Many countries face similar challenges in breeding strategies for dual-purpose chickens [6,7]. An important problem in the production of dual-purpose chickens is determining how to balance the selection for growth-related and egg-production traits [8,9]. We need more cautious breeding strategies to minimize these problems.

To address these issues, establishing a crossbreeding system for the Taiwan Country chicken industry is imperative. Crossbreeding has been implemented in local chicken breeds, demonstrating its ability to enhance environmental resilience through heterosis [10,11]. This method benefits both parent and commercial stocks [12,13]. However, the Taiwanese market has traditionally favored chickens with larger body sizes and thicker shanks. This preference has further complicated breeding objectives, as the body weight of the dam line tends to decrease with long-term selection for egg production. Therefore, traits such as body weight, shank structure, and body conformation should not only be considered in the sire line but also incorporated into the dam line to ensure that commercial meat-type chickens meet market demands.

Genomic selection, a concept introduced in 2001 [14], involves using genotypic data to predict trait performance for selection purposes. Notably, in the poultry industry, short generation intervals and large candidate populations increase genotyping costs [15], which leads to genomic selection being infrequently applied for many local chicken breeds. However, the establishment of the single-step genomic best linear unbiased prediction (ssGBLUP) model has advanced the field of genomic selection. This model considers genotyped and ungenotyped individuals in addition to their pedigree and phenotypic data to improve the accuracy of genomic estimated breeding values (GEBVs) [16]. This model has been widely adopted, with it being particularly common in the livestock breeding industry [17,18].

Because of the diversity of the markets for Taiwan Country chicken, management of the high genotyping costs associated with genomic selection of the populations must be strategic. In the present study, we aimed to explore the application of genomic prediction and marker-assisted selection (MAS) for enhancing growth-related traits in Taiwan Country chickens. We utilized both the pedigree-based best linear unbiased prediction (PBLUP) and the ssGBLUP models to estimate the accuracy of GEBVs. Furthermore, a genome-wide association study (GWAS) was conducted to identify single-nucleotide polymorphisms (SNPs) associated with growth, shank, and body conformation traits, providing a reference for future genomic selection strategies in Taiwan Country chickens.

## 2. Materials and Methods

### 2.1. Animals and Phenotyping

The animals used in this study were sourced from a purebred population of NCHU-G101 chickens, one of the Taiwan Country chicken breeds maintained as a dam line since 2007 at the experimental farm of National Chung Hsing University (NCHU) in Taichung, Taiwan. Between 2018 and 2020, three genotyped generations (G11–G13) were hatched in eight batches, with G11 serving as the base population for this study. G11 included 40 candidate roosters and 200 candidate hens, from which 10 roosters and 60 hens were selected to generate G12, consisting of 40 candidate roosters and 400 candidate hens. From G12, 15 roosters and 250 hens were selected to generate G13. Complete pedigree information was available for all 7922 chickens across these three generations. Breeding parents for each generation were selected based on their EBVs for total egg number from age at first egg to 40 weeks of age, calculated using the PBLUP model, and the next generation was produced through pedigree-based artificial insemination.

All chickens were housed in floor pens within an open-sided chicken house and provided feed ad libitum until 12 weeks of age. For generations G12 and G13, we collected data on the growth traits, including body weight at 8 weeks (BW8) and 12 weeks of age (BW12); shank traits, including shank length (SL) and shank width (SW) at 12 weeks of age; and body conformation traits, including back length (BBL) and back width (BBW) at 12 weeks of age. SL was measured using a Vernier caliper (UniSync, Taiwan) as the distance between the hock joint and the foot pad. SW was measured using a Vernier caliper as the width at the midpoint of the shank from the back to the front. BBL was measured using a measuring tape along the vertical axis from the end of the cervical vertebrae to the pygostyle. BBW was measured using a measuring tape as the longest distance between the two wings along the horizontal axis, perpendicular to the vertical axis.

### 2.2. Genotyping and Quality Control

Blood samples were collected from the wing veins of 650 chickens across the G11 to G13 generations, and genomic DNA was extracted using the Biokit Blood Genomic DNA Mini Purification Kit (Biokit, Miaoli, Taiwan). Genotyping was performed using the 600 K Affymetrix Axiom HD genotyping array (Affymetrix, Santa Clara, CA, USA), which covered 580,961 SNPs located on 30 autosomes and 2 sex chromosomes.

Quality control of the genotyping data was performed using PLINK v1.9 [19] and the BLUPF90 family software [20]. SNPs located on unknown chromosomal positions in the chicken reference genome (GRCg6a) and those on the two sex chromosomes were excluded. The data of 11 chickens were removed because the call rate was below 95%. In addition, SNPs were excluded if they had a low call frequency (<95%), deviated from the Hardy–Weinberg equilibrium (*p* < 1.00 × 10^−6^), or had a minor allele frequency of <5%. Chickens with heterozygosity exceeding ±3 standard deviations from the mean in the population were also excluded due to potential genotyping errors. Pedigree checking was performed using the seekparentf90 program (v1.49) from the BLUPF90 software, which uses Mendelian conflict analysis to validate parent–progeny relationships. This method evaluates whether the progeny’s genotype is consistent with the recorded sire and dam genotypes. Parent–progeny pairs exhibiting Mendelian conflicts were identified as inconsistent, resulting in the exclusion of 22 sires whose progeny could not be genetically confirmed.

The final dataset included 616 genotyped individuals (497 females and 119 males) with 352,429 SNPs. For genomic prediction, we applied the PBLUP model with complete pedigree and without genomic information and applied the ssGBLUP model with both genomic data from the 616 genotyped individuals and complete pedigree information. These 616 individuals from the G11 to G13 generations were among the 5185 chickens evaluated for growth traits and 4887 chickens assessed for shank and body conformation traits. Table 1 presents the descriptive statistics for the growth (BW8 and BW12), shank (SL and SW), and body conformation (BBL and BBW) traits that were considered for genomic prediction in the NCHU-G101 chickens.

### 2.3. Genomic Prediction Model

We examined growth, shank, and body conformation traits using the PBLUP and ssGBLUP models. The prediction accuracy of each model was calculated using the renumf90 (v1.161) and blupf90+ (v2.49) programs from BLUPF90 software. Variance components and genetic correlations were estimated using the expectation-maximization restricted maximum likelihood algorithm.

#### 2.3.1. PBLUP Model

The following single-trait animal model was used for data analysis:

(1) y=Xb+Za+e,

where y denotes the vector of phenotypic observations; b is the vector of fixed effects, including generation, batch, sex, and sire; a is the vector of additive genetic effects with $a\sim N\left(0,A\sigma_{a}^{2}\right)$, where A is the pedigree-based genetic relationship matrix and $\sigma_{a}^{2}$ is the variance of additive genetic effects; e is the vector of residual effects with $e\sim N\left(0,I\sigma_{e}^{2}\right)$, where I is the identity matrix and $\sigma_{e}^{2}$ is the variance of residual effects; and X and Z are the incidence matrices for the effects in b and a, respectively.

#### 2.3.2. ssGBLUP Model

In the ssGBLUP model, the A matrix in the PBLUP model is replaced with the H matrix, a hybrid relationship matrix that incorporates both pedigree and genomic relationships:

(2) y=Xb+Zu+e,

where y, b, X, Z, and e are defined as in Equation (1) and u is a vector of additive genetic effects with $u\sim N(0,H\sigma_{a}^{2})$, where H is the hybrid relationship matrix. The inverse of H is calculated using the following equation:

(3) $$H^{-1}=A^{-1}+\left[\begin{array}{cc} 0 & 0\\ 0 & \tau G^{-1}-\omega A_{22}^{-1} \end{array}\right],$$

where A is the numerator relationship matrix based on pedigree for all animals and $A_{22}$ is the relationship matrix for genotyped animals. The genomic relationship matrix G is adjusted as follows [21]: $G=\alpha G_{0}+\beta A_{22}$, where $G_{0}$ is calculated using the following:

(4) $$G_{0}=\frac{MM^{\prime }}{2\sum p_{i}(1-p_{i})}=MM\prime \lambda_{1},$$

where M is the matrix of gene content adjusted for allele frequencies, $p_{i}$ is the frequency of the *i*th SNP, and $\lambda_{1}$ is a variance ratio or a normalizing constant defined as $\frac{1}{2\sum p_{i}(1-p_{i})}$.

In this study, we used the following parameters in the ssGBLUP model: α = 0.95, β = 0.05, τ = 1.0, and ω = 1.0.

#### 2.3.3. Prediction Accuracy

The theoretical prediction accuracy (acc) was calculated on the basis of the prediction error variance (PEV), as described in Aguilar et al. [22]:

(5) $$acc=\sqrt{1-\frac{PEV}{\sigma_{a}^{2}(1+F)}},$$

where PEV is the error of variance between (G)EBVs and true genetic breeding values, $\sigma_{a}^{2}$ is the additive genetic variance, and F is the inbreeding coefficient.

### 2.4. Genome-Wide Association Study (GWAS)

Data were collected from 388, 368, and 368 individuals from the G12 and G13 generations on their growth, shank, and body conformation traits, respectively. Due to the absence of growth-related data collection in G11, the genotyped individuals from this group were excluded from subsequent analysis. We performed SNP pruning to remove linked variants and ensure a set of independent SNPs for accurate linkage disequilibrium (LD) assessment. The procedure was applied with a window size of 25 SNPs, a step size of 5 SNPs, and an r^2^ threshold of 0.2 using PLINK software (v1.9). The datasets for GWAS contained 24,989, 24,939, and 24,939 SNPs across 30 autosomes for growth, shank, and body conformation traits, respectively. Table 2 presents the descriptive statistics for GWAS in NCHU-G101 chickens.

The population structure was evaluated using principal component (PC) analysis (PCA) in PLINK software. PCs were computed, and the none PC (PC0) to top five PCs (PC5) were tested as covariates in the GWAS using GEMMA software v0.98.5 [23]. The optimal number of PCs was determined by calculating genomic inflation factors (λ), defined as the median of the observed chi-squared test statistics divided by the expected median of the chi-squared distribution. The selected PCs were then included in the GWAS analysis.

GWAS was performed using GEMMA software. Each phenotype was analyzed using a univariate linear mixed model (LMM). For each SNP, the following LMM was applied:

$$y=W\alpha +x\beta +m+\varepsilon ;m\sim {MVN}_{n}\left(0,\lambda_{2}\tau^{-1}K\right),\varepsilon \sim {MVN}_{n}\left(0,\tau^{-1}I_{n}\right),$$

where y is the vector of observations for individuals and W is the matrix of fixed effects, including generation, batch, sex, and sire. PC1 and PC2, PC1 to PC3, and PC1 to PC5 are considered to be covariates for growth, shank, and conformation traits, respectively. In addition, α is the corresponding coefficient of fixed effects; x is the vector of SNP genotypes; β is the effect size of the SNP; m is a vector of random effects; ε is the vector of residual errors; $\tau^{-1}$ is the variance of residual errors; $\lambda_{2}$ is the ratio of two variance components; K is the known relatedness matrix; $I_{n}$ is the identity matrix; and ${MVN}_{n}$ is the n-dimensional multivariate normal distribution. Significance thresholds were determined on the basis of the number of independent SNPs. For growth traits, the 5% Bonferroni genome-wide significance threshold was set at 2.00 × 10^−6^ (0.05/24,989), and the suggestive threshold was 4.00 × 10^−5^ (1.00/24,989). For shank and body conformation traits, the Bonferroni threshold was 2.00 × 10^−6^ (0.05/24,939) and the suggestive threshold was 4.01 × 10^−5^ (1.00/24,939). Manhattan and Q-Q plots were generated using the CMplot package [24] in R software v4.3.0 [25].

### 2.5. Gene Annotation and Genotype–Phenotype Association Analysis

To identify SNP genotypes and associated genes, we determined the LD block positions for each SNP that reached the 5% Bonferroni genome-wide significance or suggestive-level threshold by using the *ld* procedure in PLINK. This procedure can be used to identify all variants with an r^2^ ≥ 0.8 relative to a target SNP.

Gene annotation was performed for candidate SNPs located within LD block overlapping regions that met the 5% Bonferroni genome-wide significance or suggestive levels. BEDtools v2.30.0 [26] was used for annotation with the GRCg6a reference genome assembly. Missense SNPs and their corresponding amino acid changes were annotated using SnpEff software v5.2c [27]. To further analyze these missense SNPs, we incorporated data from the Ensembl genome browser (www.ensembl.org, accessed on 25 August 2024) and evaluated them using the sorting intolerant from tolerant (SIFT) score. SNPs with a SIFT score of ≤0.05 were classified as deleterious, indicating a potential impact on protein function.

Genotype–phenotype association analysis was performed for SNPs reaching the 5% Bonferroni genome-wide significance threshold and SNPs at the suggestive level with amino acid changes. This analysis was conducted using a general linear model in SAS software (SAS 9.4, SAS Institute Inc., Cary, NC, USA) with phenotypic data for growth, shank, and body conformation traits. The genotype effects were analyzed using the following model:

$$y_{ijklm}=\mu +Y_{i}+B_{j}+S_{k}+F_{l}+G_{m}+e_{ijklm},$$

where $y_{ijklm}$ is the phenotypic value of the analyzed trait, μ is the population mean of the analyzed trait, $Y_{i}$ is the fixed effect of generation, $B_{j}$ is the fixed effect of batch, $S_{k}$ is the fixed effect of sex, $F_{l}$ is the fixed effect of sire, $G_{m}$ is the fixed effect of the SNP genotype, and $e_{ijklm}$ is the residual effect. All values are presented as means with standard errors. Tukey’s HSD test was used to compare means for significant differences. Bar plots were generated using the ggplot2 package [28] in the R software v4.3.0.

## 3. Results

### 3.1. Genomic Prediction

The heritability estimates for each trait, derived using the PBLUP and ssGBLUP models, indicate that the inclusion of genomic information in the ssGBLUP model consistently led to higher estimates across traits (Table 3). For instance, the heritability of BW8 increased significantly from 0.370 ± 0.032 with the PBLUP model to 0.584 ± 0.049 with the ssGBLUP model. Similarly, BW12 showed an increase from 0.312 ± 0.030 to 0.508 ± 0.035. While traits such as SL and SW exhibited improvements under the ssGBLUP model, BBL and BBW demonstrated only slight increases. These findings highlight the advantage of the ssGBLUP model in providing higher heritability estimates, particularly for growth-related traits.

Table 3 also presents the genetic and phenotypic correlations between traits using the two models. Phenotypic correlations (lower-left triangular section) showed moderate to strong positive relationships among all traits. The upper-right triangular section illustrates the genetic correlations, where strong positive relationships were observed between growth traits such as BW8 and BW12 (0.986 with PBLUP; 0.989 with ssGBLUP), between BW12 and BBL (0.853 with PBLUP, 0.921 with ssGBLUP), and between BW12 and BBW (0.818 with PBLUP; 0.892 with ssGBLUP). These results indicate that the ssGBLUP model consistently provided higher estimates. In contrast, moderate genetic correlations were identified between SL and SW and between SL and BBW, suggesting relatively weaker genetic links among these traits.

The prediction accuracy of (G)EBVs for the traits analyzed in this study demonstrates the superiority of the ssGBLUP model over the PBLUP model (Table 4). For growth traits such as BW8 and BW12, the ssGBLUP model achieved significant improvements in accuracy, increasing from 0.632 ± 0.001 to 0.731 ± 0.001 for BW8 (a 15.6% increase) and from 0.605 ± 0.001 to 0.704 ± 0.001 for BW12 (a 16.4% increase). Similarly, the accuracy for SL and SW improved by 4.3% and 9.5%, respectively, under the ssGBLUP model. Body conformation traits, including BBL and BBW, also showed increases of 9.3% and 12.2%, respectively. These results highlight the effectiveness of the ssGBLUP model in predicting (G)EBVs, particularly for key growth traits such as BW8 and BW12, where the prediction accuracy was consistently higher.

### 3.2. GWAS

**Figure 1:**
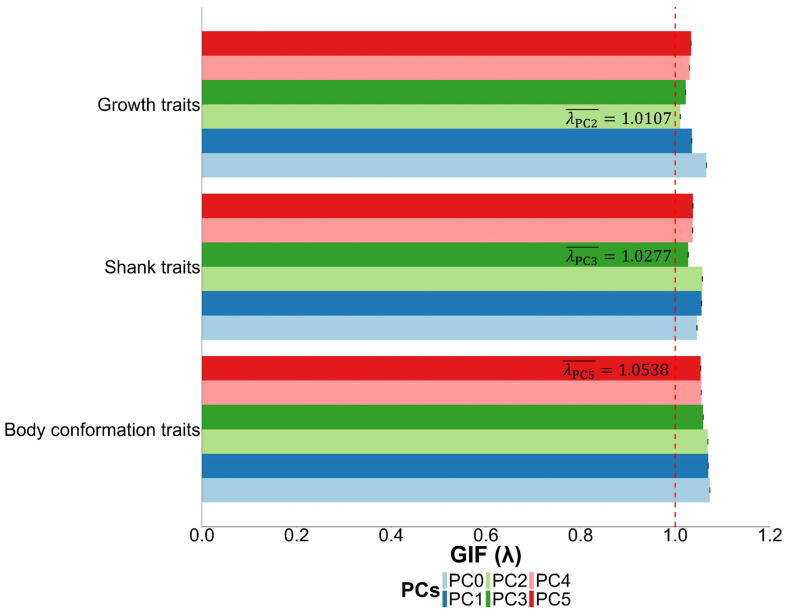
Average genomic inflation factor ($\bar{\lambda }$) when varying numbers of principal components (PCs) are used as covariates in the genome-wide association study (GWAS) for different traits. Growth traits include body weight at 8 and 12 weeks of age; shank traits include shank length and width; and body conformation traits include back length and width.

**Figure 2:**
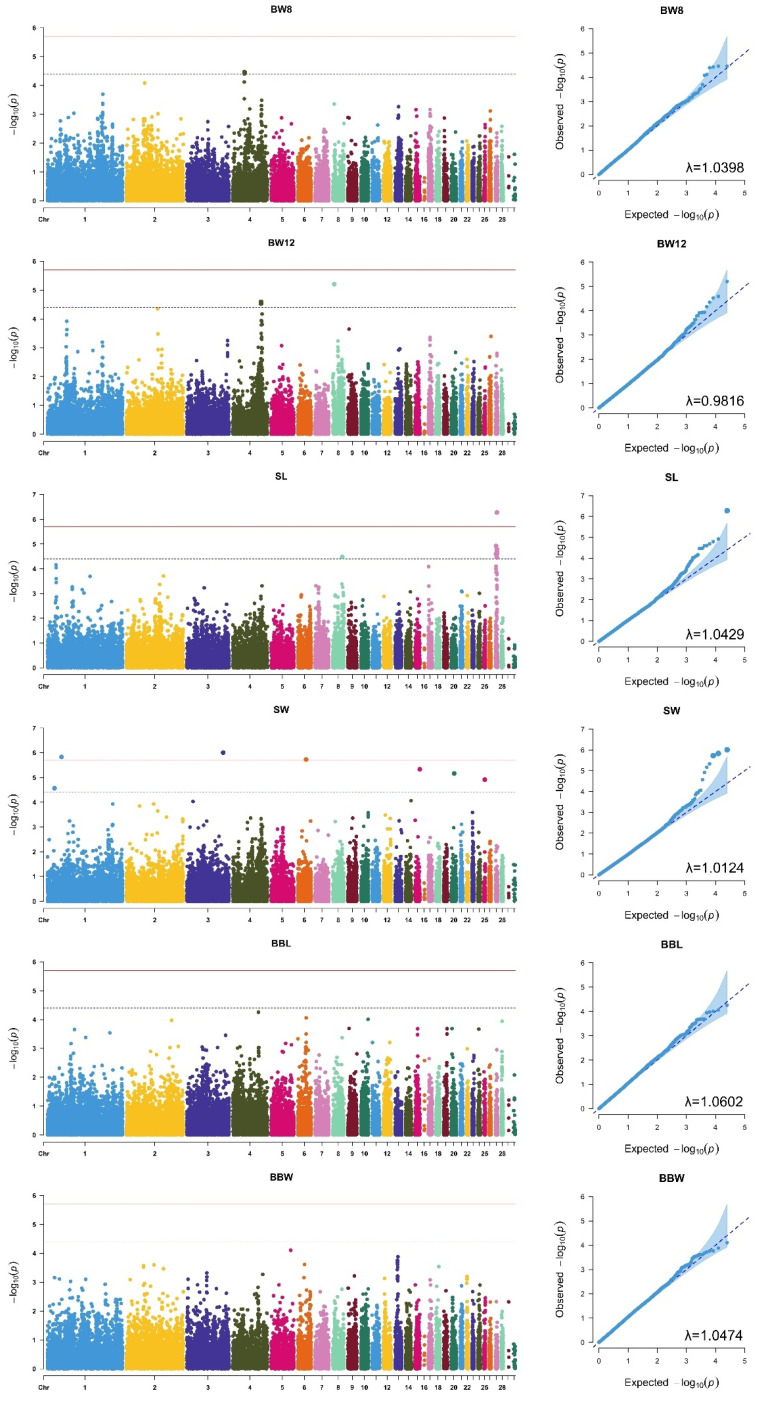
Manhattan (**left**) and QQ (**right**) plots of genome-wide association study (GWAS) results obtained using GEMMA software for body weight at 8 weeks (BW8), body weight at 12 weeks (BW12), shank length (SL), shank width (SW), back length (BBL), and back width (BBW). The red solid line represents the threshold for 5% Bonferroni genome-wide significance (*p* = 2.00 × 10^−6^ for BW8 and BW12; *p* = 2.00 × 10^−6^ for SL, SW, BBL, and BBW). The blue dotted line represents the suggestive threshold (*p* = 4.00 × 10^−5^ for BW8 and BW12; *p* = 4.01 × 10^−5^ for SL, SW, BBL, and BBW).

To decide how many principal components (PCs) to include as covariates in the GWAS model, we evaluated the genomic inflation factor (GIF, λ) for each trait across a range of PCs, from none (PC0) to the top five (Figure 1). For growth traits, including the top two PCs (PC2) yielded an average λ value closest to 1 (1.0107 ± 0.0002), indicating minimal inflation. For shank traits, including the top three PCs (PC3) yielded the optimal average λ of 1.0277 ± 0.0004, while body conformation traits achieved the most favorable λ value of 1.0538 ± 0.0001 when the top five PCs (PC5) were included. These results guided the selection of PCs for each trait category, ensuring a properly adjusted GWAS model. The Manhattan and QQ plots for each analyzed trait, generated using GEMMA software, are presented in Figure 2.

### 3.3. Findings in Gene Annotation and Genotype–Phenotype Association

The GWAS analysis identified 22 SNPs at the suggestive significance threshold, among which four surpassed the 5% Bonferroni genome-wide significance level (Figure 2). These genome-wide significant SNPs were associated with SL and SW, including AX-76362403 on GGA27, AX-75438992 on GGA1, AX-76590814 on GGA3, and AX-76920653 on GGA6. The suggestive SNPs were distributed across traits as follows: three for BW8, three for BW12, eight for SL, and four for SW. Collectively, these 22 SNPs were mapped to 14 genes, as detailed in Table 5 and Table 6, highlighting genomic regions potentially influencing growth-related traits.

For BW8, three suggestive SNPs were identified on GGA4, but no candidate genes were located at these specific positions (Table 5). However, an analysis of the LD region (30.7–31.0 Mb) revealed quantitative trait loci (QTLs) associated with body weight and shank length, suggesting underlying genetic influences. For BW12, three suggestive SNPs were identified on GGA4 and GGA8, with two located within the *PPARGC1A* gene. Among these, a missense variant, AX-80898585, in the *NOTCH2* gene on GGA8 resulted in a Pro1017Ala substitution predicted to be deleterious (Table 6).

Nine SNPs were associated with SL, located on GGA8 and GGA27, with the most significant SNP being AX-76362403 in the *ZNF652* on GGA27. Additional associations included *CDCP2*, *FMNL1*, *IGF2BP1*, *DHX8*, and *KAT7*. A missense variant, AX-80892269, in *CALCOCO2* caused a Ser85Thr substitution, which is predicted to be tolerated (SIFT score = 0.53). For SW, seven SNPs were identified across GGA1, GGA3, GGA6, GGA15, GGA20, and GGA25, with the most significant SNP being AX-76590814 on GGA3 and AX-76920653 on GGA6. Genes associated with SW included *RSAD2* and *ADGRA1*. Notably, two missense variants were identified: AX-75824016 in *RBM19* (Val400Ala) and AX-76226556 in *LAMA5* (Asp165Ala), both of which were predicted to be deleterious.

**Figure 3:**
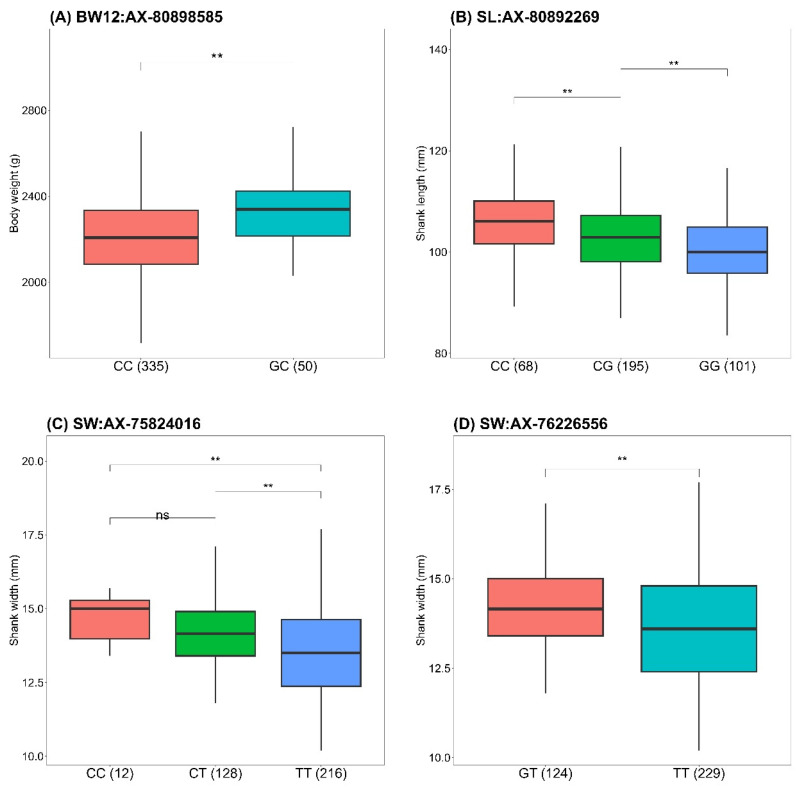
Boxplots of phenotypes for body weight at 12 weeks (BW12), shank length (SL), and shank width (SW) by alleles of missense SNP markers. The boxes show the first, median, and third quartile, with whiskers extending 1.5 times the interquartile range. ** above the bars indicates significant differences (*p* < 0.05) between genotypes within each SNP, while “ns” indicates no significant difference, based on Tukey’s HSD test. Numbers in parentheses next to the genotypes represent the sample size for each group, with the total sample size possibly being lower because of missing genotyped data for some individuals.

Genotype–phenotype associations revealed significant relationships for these missense SNPs (Table 6). For BW12, individuals with the GC genotype at AX-80898585 exhibited significantly higher body weights than those with the CC genotype (Figure 3A). For SL, individuals with the CC genotype at AX-80892269 had significantly longer SL than those with CG and GG genotypes (Figure 3B). Regarding SW, individuals with the CC and CT genotypes at AX-75824016 displayed significantly wider SW compared to those with the TT genotype (Figure 3C), while individuals with the GT genotype at AX-76226556 also had significantly wider SW than those with the TT genotype (Figure 3D).

**Figure 4:**
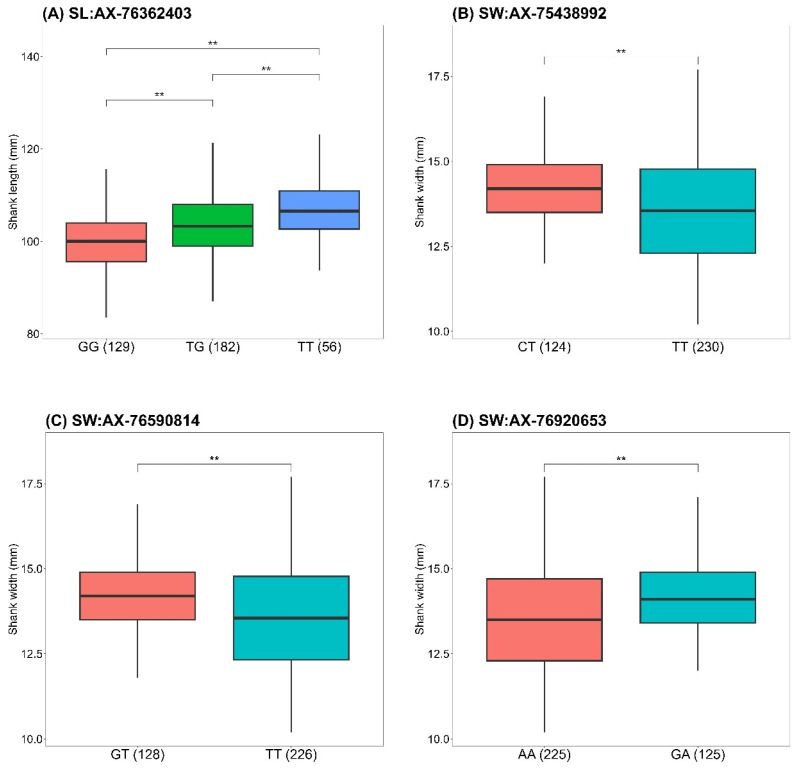
Boxplots of phenotypes for shank length (SL) and shank width (SW) by genotypes of SNP markers with 5% Bonferroni genome-wide significance. The boxes represent the first, median, and third quartiles, with whiskers extending up to 1.5 times the interquartile range. ** above the bars indicates significant differences (*p* < 0.05) based on Tukey’s HSD test. Numbers in parentheses next to the genotypes indicate the sample size for each group. The total sample size was reduced in some cases because of missing genotyped data for certain individuals.

The boxplots in Figure 4 illustrate additional genotype–phenotype associations for SNPs reaching 5% Bonferroni genome-wide significance. For SL, individuals with the TT genotype at AX-76362403 exhibited significantly longer SL compared to TG and GG genotypes (Figure 4A). For SW, significant associations were observed for the CT genotype at AX-75438992 (Figure 4B), the GT genotype at AX-76590814 (Figure 4C), and the GA genotype at AX-76920653 (Figure 4D), all of which were associated with significantly wider shanks. These results confirm strong links between these SNPs and their respective traits, particularly the missense variants in *NOTCH2*, *CALCOCO2*, *RBM19*, and *LAMA5*, which may play critical roles in growth and body conformation. Further investigation is warranted to elucidate their functional impact.

## 4. Discussion

### 4.1. Genetic Prediction Using Different Models

The heritability estimates from both the PBLUP and ssGBLUP models indicated significant genetic effects on the analyzed traits, with the ssGBLUP model consistently providing higher estimates for most traits (Table 3). The heritability of BW8 and BW12 increased when estimated using the ssGBLUP model compared to the PBLUP model. Additionally, the prediction accuracy for breeding values of BW8 and BW12 improved by 15.6% and 16.4%, respectively, when using the ssGBLUP model. For SL, the heritability in the present study ranged from 0.355 to 0.366, similar to the 0.35 reported by González-Cerón et al. [29] and Cahyadi et al. [30]. In contrast, shank width (SW) exhibited lower heritability (0.181 and 0.212), consistent with the range of 0.07 to 0.34 reported by Khurana et al. [31]. The strong relationship between heritability and prediction accuracy is evident in this study, as traits with higher heritability estimates under the ssGBLUP model also showed substantial improvements in prediction accuracy (Table 3 and Table 4). Teng et al. [32] also found that the ssGBLUP model provided higher heritability estimates and more accurate predictions in livestock populations. This finding is consistent with previous studies, which reported that the integration of genomic, pedigree, and phenotypic data in the ssGBLUP model significantly improves both heritability estimates and prediction accuracy, especially when training populations are well-structured and genetically related to the validation set [33,34]. These results demonstrate that incorporating genomic data enhances the prediction of genetic potential, particularly for growth-related traits.

In Taiwan’s poultry market, the back area of a chicken is considered a crucial commercial trait. To address this, the length and width of the chickens’ backs (BBL and BBW) were measured to estimate genetic parameters. Although these traits exhibited low heritability (Table 3), incorporating genomic data improved prediction accuracy by 9.4% and 11.4%, respectively (Table 4). This result demonstrates the effectiveness of the ssGBLUP model, even for traits with limited genetic predictability, as also reported in purebred broiler chickens [35].

Despite the lower heritability observed for some traits, the ssGBLUP model consistently provided more reliable estimates compared to the PBLUP model. The significant improvements in heritability and prediction accuracy across all traits, particularly economically important ones like body weight, highlight the model’s ability to effectively integrate genomic, pedigree, and phenotypic data. This capability allows the ssGBLUP model to enhance genetic evaluations, support better-informed selection decisions, and accelerate genetic gains in poultry breeding programs [36].

Building on these advantages, it is crucial to examine the relationships between traits to better understand how selection for one trait may influence others. Table 3 presents the high genetic correlations between BW12 and body conformation traits (BBL and BBW), estimated by the PBLUP (0.853 and 0.818) and ssGBLUP (0.921 and 0.892) models. These findings suggest that selecting for increased body weight at 12 weeks may lead to correlated genetic improvements in body conformation traits.

The genetic correlations between BBL, BBW, and growth traits highlight their close linkage and the potential influence of shared genetic factors. In contrast, their genetic correlations with SL and SW were moderate to low, suggesting that back traits and shank traits are less closely related. Lande [37] noted that genetic correlations can be maintained through selection and linkage disequilibrium, allowing simultaneous selection for multiple traits. This insight is particularly valuable for breeding programs aimed at improving body weight and body conformation traits simultaneously. In Taiwan Country chicken breeding, selecting for larger body weight not only enhances growth performance but also increases BBL and BBW, traits preferred by consumers for their economic value. These findings underscore the importance of integrating genetic correlations into selection strategies to achieve comprehensive improvements in economically important traits.

### 4.2. Genes Associated with Growth-Related Traits

Table 5 lists three SNPs associated with BW12 annotated to two genes, namely *PPARGC1A* and *NOTCH2*. The most significant SNP, AX-76727061, was associated with *PPARGC1A* on GGA4, a gene that promotes mitochondrial biogenesis and regulates skeletal muscle metabolism, both of which are linked to body weight [38]. Mitochondrial biogenesis, regulated by *PPARGC1A*, plays a critical role in optimizing energy utilization and maintaining cellular efficiency. According to Bottje [39], enhanced mitochondrial function, including increased expression of AMP-activated protein kinase (AMPK) and its catalytic subunit *PRKAA2*, activates *PPARGC1A*, which stimulates mitochondrial biogenesis and improves energy metabolism, further supporting the link between *PPARGC1A* and traits such as body weight. AX-80898585, located on GGA8, is a missense SNP associated with BW12 and annotated with *NOTCH2*. *NOTCH2* is involved in regulating the migration and proliferation of smooth muscle cells [40]. Furthermore, previous studies documented in the Chicken QTL database have shown significant associations between body weight and regions on chromosomes such as GGA4 [36], and GGA8 [41], which were also linked to growth-related genes in our study. These findings suggest that these genes are critical markers for improving body weight in poultry breeding.

For shank traits, we annotated eight SNPs on GGA27 and one SNP on GGA8 that were associated with SL, and seven SNPs on GGA1, GGA3, GGA6, GGA15, GGA20, and GGA25 that were associated with SW. Several QTLs on GGA27 have been linked to chicken body size. For SL, eight SNPs were annotated to seven genes, namely, *CDCP2*, *FMNL1*, *ZNF652*, *IGF2BP1*, *CALCOCO2*, *DHX8*, and *KAT7*. *CDCP2* is a protein-coding gene and is associated with osteosclerotic myeloma in humans [42]. *FMNL1* promotes tumorigenesis and metastasis in certain tumors and regulates asymmetric cell division by controlling actin assembly and spindle positioning in mouse oocytes [43]. AX-76362403, a genome-wide significant SNP (with a 5% Bonferroni threshold) associated with SL, is located within the intron of the *ZNF652*. Wang et al. [44] identified an association between the *ZNF652* gene on GGA27 and SL in a unique chicken intercross line. We set QTL regions based on the location of suggestive-level SNPs to detect overlap with QTLs using the ChickenQTL database. The QTL region (5.9 Mb to 6.1 Mb) surrounding AX-76362403 on GGA27 is associated with humerus length [45]. *IGF2BP1*, an mRNA-binding protein for IGF2, acts as a potent growth factor and is critical in vertebrate development [46], contributing to larger body size. Zhang et al. [47] discovered that *IGF2BP1* is associated with SL in chickens. AX-80892269, a missense SNP located on the *CALCOCO2* gene on GGA27, results in a Ser85Thr amino acid substitution. In addition, Lien et al. [48] reported that *CALCOCO2* is associated with foot weight in Taiwan Country chickens. *DHX8* gene is an RNA helicase involved in pre-mRNA splicing and post-transcriptional regulation, playing a crucial role in gene expression regulation and RNA metabolism [49]. The *KAT7* gene is a histone acetyltransferase involved in the epigenetic regulation of gene expression. In Pekin ducks and Mallard ducks, the expression levels of *KAT7* vary across different tissues, particularly playing a significant role in liver function and physiological regulation [50]. This regulatory role may indirectly influence the expression of genes associated with skeletal development, such as those involved in osteoblast differentiation [51].

For SW, four out of seven SNPs were annotated to the genes *RSAD2*, *ADGRA1*, *RBM19*, and *LAMA5* on GGA3, GGA6, GGA15, and GGA20, respectively. The most significant SNP, AX-76590814, reached the 5% Bonferroni genome-wide significance threshold and annotated to *RSAD2* on GGA3. *RSAD2* gene is an interferon-induced protein involved in the antiviral response of the innate immune system [52]. The *ADGRA1* gene is involved in the regulation of immune cells, such as macrophages, and functions as an adhesion G protein-coupled receptor [53]. It may participate in the regulation of the extracellular matrix (ECM), which is a crucial component for the development and maintenance of bones. Although there is currently no direct evidence supporting a relationship between *ADGRA1* and bone structures in the legs, such as tibial length or width, it may indirectly influence bone structure or function-related signaling pathways, including those affecting osteocyte differentiation or activity [54]. The *RBM19* gene plays a crucial role in the processing and synthesis of ribosomal RNA [55]. The primary function of the *LAMA5* gene is related to skeletal development [56]. Shank traits have been linked to QTLs on GGA6 [57]. Further exploration of previously identified QTL regions for growth and shank traits may provide valuable insights into the genetic mechanisms underlying these traits.

According to the Chicken QTL database, growth and shank traits are often associated with multiple QTL regions across various chromosomes. Ankra-Badu et al. [58], using an F2 population of broiler chickens, identified significant QTL on GGA4 that are linked to both body weight and shank traits. The QTL on GGA4 was linked to late growth (5 to 9 weeks) and had a large effect on shank traits. This polygenic nature, combined with the widespread distribution of QTLs, might dilute the signals detectable by GWAS, especially in populations with limited sample sizes [59]. Yang et al. [60] highlighted that small-effect SNPs or rare alleles often fail to meet significance thresholds in such scenarios, leading to undetected genetic variance. This challenge is particularly relevant for local chicken breeds, where limited population sizes and unique genetic structures increase the complexity of GWAS studies.

### 4.3. SIFT Score and Application of MAS

The SIFT score is a computational tool used to predict the potential impact of amino acid substitutions on protein function. This tool evaluates whether a mutation affects protein function by determining the evolutionary conservation of protein sequences. Mutations occurring in highly conserved regions are more likely to be harmful [61]. The SIFT score reflects the degree of conservation of amino acids across related proteins, with lower scores indicating a higher likelihood that the mutation will impair protein function. A score of 0 indicates a highly deleterious mutation. The use of SIFT scores in MAS enables breeders to focus on specific SNPs predicted to substantially affect traits such as growth, disease resistance, and environmental adaptation. Lipkin et al. [62] demonstrated the effectiveness of selective DNA pooling in mapping QTLs in chickens, and this approach was successfully applied to MAS, leading to improvements in production-related traits in chicken breeding programs. In the present study, we selected four missense SNPs and two SNPs that reached the 5% Bonferroni genome-wide significance threshold and analyzed their association with phenotypes and alleles. By integrating SIFT predictions with phenotypic and genotypic data, breeders can enhance the precision of their selection processes. This strategy can improve the efficiency of breeding programs by targeting beneficial genetic variations. The SNPs AX-80898585 for BW12, AX-80892269 and AX-76362403 for SL, and AX-75824016, AX-76226556, AX-75438992, AX-76590814, and AX-76920653 for SW can be incorporated into future breeding strategies for Taiwan Country chickens, specifically NCHU-G101 chicken, as a means of maintaining body weight, shank, and conformation traits.

The selected SNPs in the present study are mainly for the NCHU-G101 chicken. Because of the unique genetic background and growth characteristics of the NCHU-G101 chicken population, the findings may not be directly applicable to other breeds, which may have different genetic variations. Therefore, further validation and testing are required to confirm the relevance and effectiveness of these SNPs in other lines or breeds before they are incorporated into breeding programs.

## 5. Conclusions

The ssGBLUP model showed notable improvements in the prediction accuracy of GEBVs for all traits, with increases ranging from 4.3% to 16.4%. The greatest improvement was observed for BW12. These findings suggest that the ssGBLUP model holds the potential for enhancing prediction accuracy and genetic gain. Furthermore, this model allows the implementation of genomic selection without the need to genotype all individuals, making it feasible for small populations to benefit from genomic selection strategies. In the GWAS, four missense SNPs and four SNPs surpassing the 5% Bonferroni genome-wide significance threshold were identified, showing associations with BW12, SL, and SW. These SNPs could be valuable for MAS in chicken breeding. The integration of the ssGBLUP model with MAS may help to accelerate genetic improvement in Taiwan Country chickens, particularly in NCHU-G101 chickens. Nonetheless, additional research is needed to further validate these results, explore their mechanisms and broader applicability, and assess their long-term impact on breeding programs.

## Figures and Tables

**Table 1:** Descriptive statistics of the genomic prediction model for growth, shank, and body conformation traits.

Trait	Sex	N	Mean	SD	Min	Max	CV (%)
Growth traits							
BW8 (g)	Male	2605	1467	216	644	2031	14.7
	Female	2580	1196	191	611	1814	16.0
	Pooled	5185	1332	245	611	2031	18.4
BW12 (g)	Male	2605	2366	333	1169	3209	14.1
	Female	2580	1864	293	943	2796	15.7
	Pooled	5185	2116	401	943	3209	19.0
Shank traits							
SL (mm)	Male	2495	118.2	6.7	88	142	5.7
	Female	2392	96.7	8.9	70	131	9.2
	Pooled	4887	107.7	13.3	70	142	12.3
SW (mm)	Male	2495	15.3	1.3	11	19	8.4
	Female	2392	13.0	1.3	10	19	10.1
	Pooled	4887	14.2	1.7	10	19	12.1
Body conformation traits							
BBL (mm)	Male	2495	241.2	16.7	185	285	6.9
	Female	2392	223.7	14.0	182	274	6.3
	Pooled	4887	232.6	17.8	182	285	7.6
BBW (mm)	Male	2495	161.2	13.4	113	200	8.3
	Female	2392	146.7	13.3	109	200	9.1
	Pooled	4887	154.1	15.2	109	200	9.9

SD, standard deviation; Min, minimum value; Max, maximum value; BW8, body weight at 8 weeks of age; BW12, body weight at 12 weeks of age; SL, shank length; SW, shank width; BBL, back length; BBW, back width.

**Table 2:** Descriptive statistics of GWAS for growth, shank, and body conformation traits.

Trait	Sex	N	Mean	SD	Min	Max	CV (%)
Growth traits							
BW8 (g)	Male	40	1742	75	1463	1876	4.3
	Female	348	1387	112	1046	1814	8.0
	Pooled	388	1423	153	1046	1876	10.8
BW12 (g)	Male	40	2787	90	2602	2962	3.2
	Female	348	2208	156	1716	2796	7.1
	Pooled	388	2268	232	1716	2962	10.2
Shank traits							
SL (mm)	Male	44	121.5	4.3	111	128	3.5
	Female	324	101.4	5.7	82	118	5.6
	Pooled	368	103.8	8.6	82	128	8.2
SW (mm)	Male	44	15.8	1.0	13	18	6.1
	Female	324	13.6	1.3	10	18	9.5
	Pooled	368	13.9	1.5	10	18	10.4
Body conformation traits							
BBL (mm)	Male	44	253.6	11.0	225	273	4.4
	Female	324	231.0	12.4	195	263	5.4
	Pooled	368	233.7	14.3	195	273	6.1
BBW (mm)	Male	44	170.2	12.1	135	194	7.1
	Female	324	153.0	12.4	115	186	8.1
	Pooled	368	155.1	13.6	115	194	8.8

SD, standard deviation; Min, minimum value; Max, maximum value; BW8, body weight at 8 weeks of age; BW12, body weight at 12 weeks of age; SL, shank length; SW, shank width; BBL, back length; BBW, back width.

**Table 3:** Heritability ± SE and phenotypic and genetic correlations ± SE for analyzed traits across different models ^1^.

	PBLUP						ssGBLUP					
Trait	BW8	BW12	SL	SW	BBL	BBW	BW8	BW12	SL	SW	BBL	BBW
BW8	0.370 ± 0.032	0.986 ± 0.008	0.569 ± 0.047	0.810 ± 0.041	0.796 ± 0.044	0.811 ± 0.051	0.584 ± 0.049	0.989 ± 0.006	0.664 ± 0.053	0.838 ± 0.045	0.867 ± 0.046	0.868 ± 0.051
BW12	0.858	0.312 ± 0.030	0.587 ± 0.046	0.795 ± 0.043	0.853 ± 0.038	0.818 ± 0.049	0.858	0.508 ± 0.035	0.690 ± 0.049	0.823 ± 0.047	0.921 ± 0.038	0.892 ± 0.045
SL	0.739	0.801	0.355 ± 0.031	0.675 ± 0.052	0.677 ± 0.054	0.484 ± 0.072	0.739	0.801	0.366 ± 0.046	0.756 ± 0.059	0.761 ± 0.061	0.588 ± 0.083
SW	0.538	0.634	0.653	0.181 ± 0.035	0.669 ± 0.068	0.568 ± 0.080	0.538	0.634	0.653	0.212 ± 0.026	0.728 ± 0.079	0.623 ± 0.090
BBL	0.640	0.682	0.642	0.502	0.140 ± 0.022	0.763 ± 0.065	0.640	0.682	0.642	0.502	0.154 ± 0.034	0.828 ± 0.070
BBW	0.526	0.540	0.518	0.460	0.539	0.135 ± 0.031	0.526	0.540	0.518	0.460	0.539	0.165 ± 0.034

PBLUP, pedigree-based best linear unbiased prediction; ssGBLUP, single-step genomic best linear unbiased prediction; SE, standard error; BW8, body weight at 8 weeks of age; BW12, body weight at 12 weeks of age; SL, shank length; SW, shank width; BBL, back length; BBW, back width. ^1^ The upper-right triangular portion of the matrix presents genetic correlations, whereas the lower-left triangular portion displays phenotypic correlations. The diagonal elements represent heritability. Genetic correlations were estimated using the PBLUP and ssGBLUP models, with the EM-REML algorithm implemented in BLUPF90 software. Phenotypic correlations are presented as Pearson correlation coefficients (n = 5185 for BW8 and BW12; n = 4887 for SL, SW, BBL, and BBW).

**Table 4:** Prediction accuracy ± SE of genomic estimated breeding values for analyzed traits using the PBLUP and ssGBLUP models.

Trait	PBLUP	ssGBLUP	Accuracy Increase
BW8	0.632 ± 0.001 ^b^	0.731 ± 0.001 ^a^	15.6%
BW12	0.605 ± 0.001 ^b^	0.704 ± 0.001 ^a^	16.4%
SL	0.618 ± 0.001 ^b^	0.644 ± 0.001 ^a^	4.3%
SW	0.519 ± 0.001 ^b^	0.569 ± 0.001 ^a^	9.5%
BBL	0.483 ± 0.001 ^b^	0.528 ± 0.001 ^a^	9.3%
BBW	0.478 ± 0.001 ^b^	0.537 ± 0.001 ^a^	12.2%

PBLUP, pedigree-based best linear unbiased prediction; ssGBLUP, single-step genomic best linear unbiased prediction; SE, standard error; BW8, body weight at 8 weeks of age; BW12, body weight at 12 weeks of age; SL, shank length; SW, shank width; BBL, back length; BBW, back width. ^a,b^ different letters above the values indicate significant differences between the models within each trait based on Tukey’s HSD test (*p* < 0.05).

**Table 5:** Annotations of 5% Bonferroni genome-wide significance and suggestive-level SNPs in GWAS.

Trait	SNP	GGA	Position	REF	ALT	*p* Value	Gene	Consequence (kb)
BW8	AX-76641120	4	30,698,290	G	A	3.48 × 10^−5^	*NA*	Intergenic variant
BW8	AX-76641728	4	31,056,795	A	G	3.48 × 10^−5^	*NA*	Intergenic variant
BW8	AX-76641316	4	30,819,031	G	A	3.78 × 10^−5^	*NA*	Intergenic variant
BW12	AX-76727061	4	74,196,434	T	C	2.58 × 10^−5^	*PPARGC1A*	Intergenic variant (D95.73)
BW12	AX-76726868	4	74,095,744	G	A	2.98 × 10^−5^	*PPARGC1A*	Intron variant
BW12	AX-80898585	8	4,602,740	G	C	6.26 × 10^−6^	*NOTCH2*	Missense variant
SL	AX-77093256	8	25,497,082	A	G	3.33 × 10^−5^	*CDCP2*	Intron variant
SL	AX-76362403 *	27	6,006,536	T	G	5.30 × 10^−7^	*ZNF652*	Intron variant
SL	AX-76353306	27	3,826,956	G	A	1.20 × 10^−5^	*FMNL1*	Intron variant
SL	AX-76362671	27	6,057,305	A	G	1.62 × 10^−5^	*IGF2BP1*	Intron variant
SL	AX-80892269	27	6,120,859	C	G	2.08 × 10^−5^	*CALCOCO2*	Missense variant
SL	AX-76362262	27	5,977,273	A	G	2.51 × 10^−5^	*DHX8*	Intron variant
SL	AX-76353023	27	3,758,771	T	C	2.52 × 10^−5^	*FMNL1*	Downstream gene variant (D16.54)
SL	AX-76360816	27	5,682,417	T	C	3.47 × 10^−5^	*NA*	Intergenic variant
SL	AX-76360955	27	5,711,610	G	A	3.47 × 10^−5^	*KAT7*	Synonymous variant
SW	AX-75438992 *	1	37,356,730	C	T	1.48 × 10^−6^	*NA*	Intergenic variant
SW	AX-80957999	1	18,899,763	C	G	2.71 × 10^−5^	*NA*	Intergenic variant
SW	AX-76590814 *	3	94,995,017	G	T	9.84 × 10^−7^	*RSAD2*	Intron variant
SW	AX-76920653 *	6	22,746,154	G	A	1.88 × 10^−6^	*ADGRA1*	Intron variant
SW	AX-75824016	15	12,747,357	C	T	4.66 × 10^−6^	*RBM19*	Missense variant
SW	AX-76226556	20	7,878,950	G	T	6.85 × 10^−6^	*LAMA5*	Missense variant
SW	AX-80776394	25	2,435,069	T	A	1.21 × 10^−5^	*NA*	Downstream gene variant

BW8, body weight at 8 weeks of age; BW12, body weight at 12 weeks of age; SL, shank length; SW, shank width; GGA, *Gallus gallus* chromosome; REF, reference allele; ALT, alternative allele; NA, not available; D, indicates that the SNP is located downstream of a gene; * The 5% Bonferroni genome-wide significant SNP.

**Table 6:** Characterization of missense SNPs annotated in candidate genes associated with analyzed traits in chickens.

Gene	SNP	GGA	Position (bp)	Trait	Amino Acid Change	SIFT Score
*NOTCH2*	AX-80898585	8	4,602,740	BW12	Pro1017Ala	Deleterious (0)
*CALCOCO2*	AX-80892269	27	6,120,859	SL	Ser85Thr	Tolerated (0.53)
*RBM19*	AX-75824016	15	12,747,357	SW	Val400Ala	Deleterious (0)
*LAMA5*	AX-76226556	20	7,878,950	SW	Asp165Ala	Deleterious (0)

GGA, *Gallus gallus* chromosome; BW12, body weight at 12 weeks of age; SL, shank length; SW, shank width.

## Data Availability

The datasets analyzed in this study are available from the corresponding author upon reasonable request.
